# Geographic variations in lipid-lowering therapy utilization, LDL-C levels, and proportion retrospectively meeting the ACC/AHA very high-risk criteria in a real-world population of patients with major atherosclerotic cardiovascular disease events in the United States

**DOI:** 10.1016/j.ajpc.2021.100177

**Published:** 2021-03-30

**Authors:** Seth J. Baum, Pallavi B. Rane, Sasikiran Nunna, Mohdhar Habib, Kiran Philip, Kainan Sun, Xin Wang, Rolin L. Wade

**Affiliations:** a Department of Integrated Medical Sciences, Charles E. Schmidt College of Medicine, Florida Atlantic University, Boca Raton, FL USA; b Amgen Inc., Thousand Oaks, CA USA; c IQVIA, Plymouth Meeting, PA USA

**Keywords:** Atherosclerotic cardiovascular disease, Very high-risk, LDL-C, Lipid-lowering therapy

## Abstract

**Objective:**

We assessed national- and state-level geographic variations among patients with a history of ≥1 major atherosclerotic cardiovascular disease (ASCVD) event in: (1) the proportion of patients with retrospectively identified 2018 American College of Cardiology/American Heart Association guideline very high-risk (VHR) ASCVD criteria; (2) utilization of guideline-directed lipid-lowering therapy (LLT); and (3) the proportion of patients with persistent low-density lipoprotein cholesterol (LDL-C) elevations despite statin and/or ezetimibe use.

**Methods:**

A retrospective cohort study using the Prognos LDL-C database linked to IQVIA longitudinal medical and prescription claims databases. The study period was from January 01, 2011, to November 30, 2019 and the index period was from January 01, 2016, to November 30, 2019; the index date was defined as the most recent LDL-C test during the index period. The study included patients aged ≥18 years at index who had a measured LDL-C level during the index period and had ≥1 inpatient/outpatient claim for ASCVD during the 5-year pre-index period.

**Results:**

Of patients with any ASCVD (N=4652,468), 1537,514 (33.1%) patients had ≥1 major ASCVD event. Among patients with ≥1 major ASCVD event, the VHR ASCVD criteria were retrospectively identified in 1139,018 (74.1%) patients; Hawaii had the highest (81.7%) and Colorado the lowest (65.0%) proportion of these patients. Nationally, 48.8% and 50.2% of patients with ≥1 major ASCVD event and retrospectively identified VHR ASCVD criteria, respectively, had current LLT use; Massachusetts and Colorado had the highest and lowest proportions, respectively. After standardizing for age and sex, 57.3% and 58.8% of patients with ≥1 major ASCVD event and retrospectively identified VHR ASCVD criteria, respectively, had LDL-C ≥70 mg/dL (≥1.8 mmol/L) despite statin and/or ezetimibe use, with substantial state-level variations observed.

**Conclusions:**

The study highlights high rates of elevated LDL-C and pervasive underuse of LLT in health-insured patients with a history of major ASCVD events treated in the United States, with state-level geographic variations observed.

**Figure:**
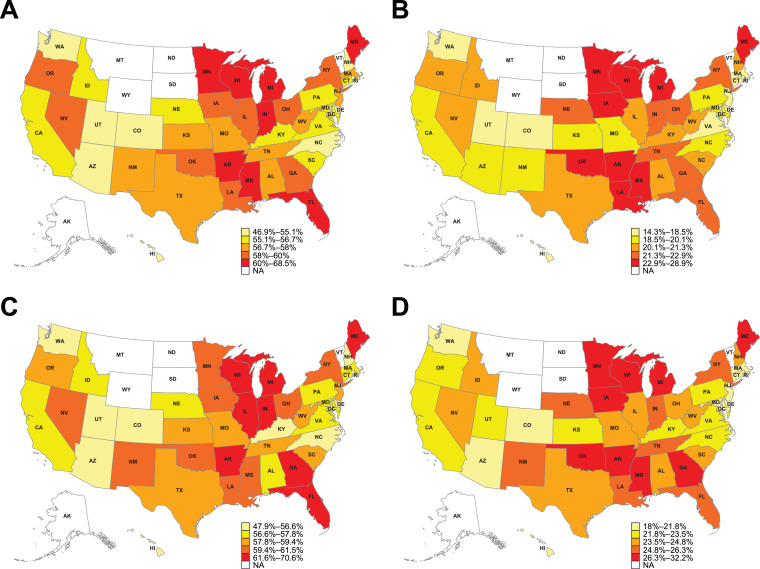
Central Illustration. Heatmap of (A) LDL-C ≥70 mg/dL (≥1.8 mmol/L) despite current statin and/or ezetimibe use in patients with a history of ≥1 major ASCVD event; (B) LDL-C ≥100 mg/dL (≥2.6 mmol/L) despite current statin and/or ezetimibe use in patients with a history of ≥1 major ASCVD event; (C) LDL-C ≥70 mg/dL (≥1.8 mmol/L) despite current statin and/or ezetimibe use in patients with retrospectively identified VHR ASCVD criteria; (D) LDL-C ≥100 mg/dL (≥2.6 mmol/L) despite current statin and/or ezetimibe use in patients with retrospectively identified VHR ASCVD criteria. Data are age- and sex-standardized percentages. Due to low sample sizes, values for Alaska, Montana, North Dakota, South Dakota, Vermont, and Wyoming are masked. ASCVD, atherosclerotic cardiovascular disease; LDL-C, low-density lipoprotein cholesterol; NA, not available; VHR, very high-risk.

## 1 Introduction

Cardiovascular disease remains the leading cause of death in the United States (US) [1], with a combined annual direct and indirect cost burden of $555 billion, which is predicted to rise to $1.1 trillion from 2015 to 2035 [2]. Low-density lipoprotein cholesterol (LDL-C) is a well-established, causal risk factor for atherosclerotic cardiovascular disease (ASCVD) [3,4]. Accordingly, lowering of LDL-C with lipid-lowering therapy (LLT) significantly reduces the risk of cardiovascular events [5]. Recent updates in the 2018 American College of Cardiology/American Heart Association (ACC/AHA) Multisociety blood cholesterol guidelines introduced the very high-risk (VHR) ASCVD classification [6]. Patients in this VHR group have multiple major ASCVD events (i.e. recent acute coronary syndrome [ACS], history of myocardial infarction [MI] other than ACS, history of ischemic stroke [IS], or symptomatic peripheral arterial disease [PAD]), or a single major ASCVD event and a history of multiple high-risk conditions [6]. In patients with VHR ASCVD and LDL-C ≥70 mg/dL (≥1.8 mmol/L) despite high-intensity or maximally tolerated statin therapy, the addition of non-statin therapy (ezetimibe and/or a proprotein convertase subtilisin/kexin type 9 inhibitor [PCSK9i]) is recommended [6].

Previous studies of US clinical practice have reported suboptimal utilization of guideline-directed LLT for patients with hypercholesterolemia, with poor statin adherence, underdosing and/or discontinuation frequently observed [7], [8], [9], [10], [11], [12], [13], [14], [15]. However, the clinical and treatment characteristics of patients at the highest cardiovascular risk (e.g. those with a history of ≥1 major ASCVD event) remain to be fully elucidated in a real-world setting. Additionally, given the recent introduction of the VHR ASCVD risk stratification in the 2018 ACC/AHA blood cholesterol guidelines, [6] the proportion of patients meeting these criteria in real-world, US clinical practice remains largely unknown. Importantly, the provision of guideline-directed LLT to reduce cardiovascular risk in patients with major ASCVD events should be uniform across the US; however, to date, VHR ASCVD status, LLT utilization, and LDL-C levels in patients with major ASCVD events have not been assessed at the state level in the US. This is an important gap in the literature, with assessment at the state level required to identify potential heterogeneity in the treatment patterns, clinical outcomes, and cardiovascular risk status of patients with major ASCVD events. Moreover, state-level analysis allows for the generalizability of national trends to be assessed, and the uncovering of disparities can inform subsequent public health interventions and policies [16].

Therefore, the objectives of the current study were to describe US national- and state-level geographic variations among patients with a history of ≥1 major ASCVD event with regard to the proportion of patients with retrospectively identified 2018 ACC/AHA guideline VHR ASCVD criteria, [6] utilization of guideline-directed LLT, and the proportion of patients with persistent LDL-C elevations despite statin and/or ezetimibe use.

## 2 Methods

### 2.1 Study design and databases

This was a retrospective cohort study using the nationally representative Prognos LDL-C database (Prognos, New York, NY, USA) [17] linked to anonymized longitudinal medical and prescription claims data across IQVIA (Plymouth Meeting, PA, USA) data sources [18]. Prognos data aggregates LDL-C values from large national and regional laboratory providers in the US, and the Prognos Registry is the largest source of clinical diagnostics information in 35 disease areas, with over 8 billion medical records for 150 million patients. The IQVIA Longitudinal Prescription claims (LRx) database captures information on adjudicated dispensed prescriptions sourced from retail, mail, long-term care, and specialty pharmacies, and represents 86% of prescriptions dispensed in retail pharmacies, 55% of prescriptions dispensed by standard mail service, along with 40%–70% of specialty pharmacy volume. Being a pharmacy claims database, the IQVIA LRx database does not contain clinical and diagnostic information. Thus, to capture clinical characteristics, the IQVIA LRx database was linked to the IQVIA PharMetrics® Plus health plan claims database and the IQVIA Professional Fee Claims database (Dx). The aggregated IQVIA PharMetrics Plus database comprises adjudicated claims for more than 130 million unique patients across the US and is sourced directly from insurance companies. IQVIA PharMetrics Plus data have a diverse representation of geography, employers, payers, providers, and therapy areas. The IQVIA Dx database contains unadjudicated medical claims from office-based physicians, ambulatory facilities, and hospital-based physicians, and is sourced from clearing houses (also referred to as switches) involved in claims processing. The study utilized de-identified health claims data and thus was exempt from institutional review board review.

The overall study period was from January 01, 2011, to November 30, 2019. The index period was from January 01, 2016, to November 30, 2019, to allow for at least a 5-year pre-index period, with the index date defined as the most recent LDL-C test during the index period (Supplementary Fig. 1). No minimum post-index follow-up was required. The 5-year pre-index period was used to capture the patient's ASCVD status, history of risk factors, and major ASCVD events among all patients, to identify those who retrospectively met the VHR ASCVD criteria per the updated ACC/AHA guidelines published in November 2018 during the index period of the current study [6] (Supplementary Fig. 1; Supplementary Table 1).

Patients across the different IQVIA databases and the Prognos LDL-C dataset were linked to create a final cohort using a Health Insurance Portability and Accountability Act (HIPAA)–compliant encrypted ID. The study complied with all applicable laws regarding patient privacy, using HIPAA-compliant de-identified retrospective data sources. No direct patient contact or primary collection of individual patient data occurred. Study results were in tabular form and aggregate analyses that omitted patient identification.

### 2.2 Patients and outcomes

The study included patients aged ≥18 years at index, who had a measured LDL-C level during the index period and had ≥1 inpatient/outpatient claim for ASCVD during the 5-year pre-index period identified using International Classification of Diseases (ICD)-9, ICD-10, and/or Current Procedural Terminology codes.

From all patients identified with ASCVD, the proportion of patients with a history of ≥1 major ASCVD event(s) and the proportion of patients with retrospectively identified 2018 ACC/AHA criteria for VHR ASCVD in the 5-year pre-index period were estimated at the national and state levels [6]. While state-level data were calculated and reported for all states, while ranking, only states with a sample size in the top 90th percentile were considered (states with a sample size in the bottom 10th percentile were not ranked).

Per the 2018 ACC/AHA guidelines [6], major ASCVD events were defined as the presence of recent ACS (within the past 12 months), history of MI (other than a recent ACS event), history of IS, or symptomatic PAD, identified using ICD-9/ICD-10 codes. Per these guidelines [6], VHR ASCVD was retrospectively defined in the current study as a history of multiple major ASCVD events, or 1 major ASCVD event and a history of multiple high-risk conditions during the 5-year pre-index period. The detailed criteria and associated operational definitions, including diagnostic codes, for major ASCVD events and high-risk conditions used in the current study are reported in Supplementary Table 1.

LDL-C distribution of the patients was assessed using the most recent LDL-C value measured on the index date. Current LLT use was estimated at the national and state levels, with patients classed as in receipt of current LLT if they received any statin, ezetimibe, or PCSK9i therapy on the index date or during the 3 months prior to the index date. National-level estimates for LLT use on the index date or during the 12 months prior to the index date were also calculated for all patient groups. The following were the categories of LLT use: “PCSK9i use (monotherapy or in combination with statins)”; “statin only”; “statin plus ezetimibe”; and “ezetimibe only”.

Age- and sex-standardized rates of patients with a history of ≥1 major ASCVD event, patients with retrospectively identified VHR ASCVD criteria per the 2018 ACC/AHA guidelines [6], current LLT use, and patients with elevated LDL-C (≥70 mg/dL [≥1.8 mmol/L] and ≥100 mg/dL [≥2.6 mmol/L]) despite current statin and/or ezetimibe use in the 2 ASCVD subgroups were reported. These were calculated by using the age and sex distribution of a standard population of patients with ASCVD in the US. A standard population of patients with ASCVD was used for age and sex standardization as opposed to census population estimates because the age distribution of patients with ASCVD is different from the age distribution of the general population in the US; thus, the census population cannot be considered a standard population [19]. Prevalence estimates of the standard ASCVD population by state and across each age group and sex category in the US were computed in a separate sample of patients who were enrolled in the IQVIA PharMetrics Plus database during 2017. These prevalence rates across each age group and sex category were then applied to the most recent census data (2017) available at the time of the analysis to obtain the final standard population to be used for the purpose of this study [19,20]. Directly standardized rates were calculated using the following formula: directly standardized rate = (r_1_N_1_ + r_2_N_2_ + r_3_N_3_ + …+ r_n_N_n_) / (N_1_ + N_2_ + N_3_ +…+ N_n_), where r_k_ equals the rate in k-th stratum of study sample (stratum refers to each age group category by sex) and N_k_ equals the number of persons in k-th stratum of the standard population.

### 2.3 Statistical analysis

Analyses were conducted using SAS version 9.3 (SAS Institute, Cary, NC, USA). The study was descriptive in nature and formal statistical tests were not conducted. Mean, median, and standard deviation (SD) were generated as measures of central tendency and variance for continuous variables. Frequencies and percentages were calculated for categorical variables.

## 3 Results

### 3.1 Baseline characteristics

From a total population of 33,910,626 patients with a measured LDL-C level in the linked database during the index period, the study included 4652,468 patients with a history of any ASCVD in the 5-year pre-index period. The mean (SD) age of the ASCVD patients was 70.0 (11.8) years and 52.6% were male; their mean (SD) baseline LDL-C was 90.8 (35.3) mg/dL (2.4 [0.9] mmol/L). The majority of patients (56.5%) were insured by a commercial payer, and the Southern region of the US had the highest proportion (51.5%) of patients with ASCVD (Table 1). Of patients with any ASCVD, 1537,514 (33.1%) patients had a history of ≥1 major ASCVD event in the 5-year pre-index period (Table 1). The mean (SD) LDL-C in patients with a history of ≥1 major ASCVD event was 87.2 (35.6) mg/dL (2.3 [0.9] mmol/L); additional patient clinical characteristics are reported in Table 2.

**Table 1:** Baseline demographic characteristics.

**Demographics**	**Patients with a history of ASCVD (N=4652,468)**	**Patients with a history of ≥1 major ASCVD event (N=1537,514)**	**Patients with retrospectively identified VHR ASCVD criteria (N=1139,018)**
Age, years			
Mean ± SD	70.0 ± 11.8	69.8 ± 12.1	71.0 ± 11.3
Median (min, max)	71 (18, 119)	71 (18, 119)	72 (18, 119)
Age group, years, N (%)			
18–34	42,481 (0.9)	14,068 (0.9)	5513 (0.5)
35–44	96,669 (2.1)	34,453 (2.2)	18,135 (1.6)
45–54	332,144 (7.1)	119,093 (7.8)	72,745 (6.4)
55–64	890,908 (19.2)	305,829 (19.9)	199,896 (17.6)
65+	3290,266 (70.7)	1064,071 (69.2)	842,729 (74.0)
Sex, N (%)			
Male	2446,291 (52.6)	846,922 (55.1)	622,203 (54.6)
Geographic region, N (%)			
Northeast	963,084 (20.7)	297,772 (19.4)	215,266 (18.9)
Midwest	489,360 (10.5)	172,428 (11.2)	131,773 (11.6)
South	2393,943 (51.5)	793,764 (51.6)	590,291 (51.8)
West	806,068 (17.3)	273,545 (17.8)	201,685 (17.7)
Unknown	13 (0.0)	5 (0.0)	3 (0.0)
Payer type, N (%)			
Commercial	2630,016 (56.5)	877,517 (57.1)	618,654 (54.3)
Medicare	1984,505 (42.7)	644,005 (41.9)	509,195 (44.7)
Other	37,947 (0.8)	15,992 (1.0)	11,169 (1.0)

ASCVD, atherosclerotic cardiovascular disease; SD, standard deviation; VHR, very high-risk.

**Table 2:** Clinical characteristics.

	**Patients with a history of ASCVD (N=4652,468)**	**Patients with a history of ≥1 major ASCVD event (N=1537,514)**	**Patients with retrospectively identified VHR ASCVD criteria (N=1139,018)**
Index LDL-C, mg/dL*			
Mean ± SD	90.8 ± 35.3	87.2 ± 35.6	87.3 ± 36.1
Median (min, max)	85 (10, 495)	81 (10, 492)	81 (10, 492)
Index LDL-C, mmol/L*			
Mean ± SD	2.4 ± 0.9	2.3 ± 0.9	2.3 ± 0.9
Median (min, max)	2.2 (0.3, 12.8)	2.1 (0.3, 12.7)	2.1 (0.3, 12.7)
Index LDL-C group, N (%)*			
<70 mg/dL (<1.8 mmol/L)	1384,265 (29.8)	536,532 (34.9)	401,443 (35.2)
70–99 mg/dL (1.8–2.6 mmol/L)	1652,824 (35.5)	530,122 (34.5)	381,044 (33.5)
100–129 mg/dL (2.6–3.3 mmol/L)	982,622 (21.1)	284,050 (18.5)	215,636 (18.9)
130–189 mg/dL (3.4–4.9 mmol/L)	578,443 (12.4)	168,924 (11.0)	126,628 (11.1)
>189 mg/dL (>4.9 mmol/L)	54,314 (1.2)	17,886 (1.2)	14,267 (1.3)
Major ASCVD events, N (%)			
Any major events	1537,514 (33.1)	1537,514 (100.0)	1139,018 (100.0)
Recent ACS	153,552 (3.3)	153,552 (10.0)	126,380 (11.1)
History of MI (other than recent ACS)	785,493 (16.9)	785,493 (51.1)	579,807 (50.9)
History of IS	675,284 (14.5)	675,284 (43.9)	488,780 (42.9)
Symptomatic PAD	132,921 (2.9)	132,921 (8.7)	104,190 (9.2)
High-risk conditions, N (%)			
Age ≥65 years	675,136 (14.5)	215,016 (14.0)	179,253 (15.7)
HeFH	132,820 (2.9)	45,629 (3.0)	38,554 (3.4)
History of prior CABG or PCI outside of major ASCVD event(s)	446,361 (9.6)	249,269 (16.2)	222,732 (19.6)
Diabetes mellitus	658,357 (14.2)	235,760 (15.3)	209,093 (18.4)
Hypertension	1856,315 (39.9)	671,120 (43.7)	612,348 (53.8)
Chronic kidney disease (eGFR 15–59 mL/min/1.73 m^2^)	479,358 (10.3)	195,907 (12.7)	160,364 (14.1)
Current smoking	435,124 (9.4)	184,794 (12.0)	162,936 (14.3)
Persistently elevated LDL-C ≥100 mg/dL despite maximally tolerated statin and ezetimibe	993,948 (21.4)	305,957 (19.9)	254,908 (22.4)
History of CHF	644,261 (13.9)	313,218 (20.4)	267,979 (23.5)
LLT use, N (%)			
Any LLT (12-month pre-index)	2712,632 (58.3)	991,624 (64.5)	758,625 (66.6)
Statin only	2524,665 (93.1)	924,992 (93.3)	708,742 (93.4)
High-intensity statin	941,126 (34.7)	424,052 (42.8)	323,448 (42.6)
Medium-intensity statin	1368,336 (50.4)	437,124 (44.1)	334,918 (44.2)
Low-intensity statin	215,203 (7.9)	63,816 (6.4)	50,376 (6.6)
Statin + ezetimibe	127,076 (4.7)	45,475 (4.6)	33,830 (4.5)
High-intensity statin	67,383 (2.5)	26,699 (2.7)	19,508 (2.6)
Medium-intensity statin	52,580 (1.9)	16,563 (1.7)	12,599 (1.7)
Low-intensity statin	7113 (0.3)	2213 (0.2)	1723 (0.2)
Ezetimibe only	38,898 (1.4)	12,034 (1.2)	9487 (1.3)
PCSK9i (monotherapy or in combination with statin)	21,993 (0.8)	9123 (0.9)	6566 (0.9)
Current LLT (3-month pre-index)	2044,686 (43.9)	749,902 (48.8)	571,972 (50.2)
Statin only	1903,687 (93.1)	700,059 (93.4)	534,965 (93.5)
High-intensity statin	694,927 (34.0)	316,748 (42.2)	241,383 (42.2)
Medium-intensity statin	1037,293 (50.7)	332,718 (44.4)	253,897 (44.4)
Low-intensity statin	171,467 (8.4)	50,593 (6.7)	39,685 (6.9)
Statin + ezetimibe	74,430 (3.6)	26,421 (3.5)	19,453 (3.4)
High-intensity statin	39,645 (1.9)	15,561 (2.1)	11,215 (2.0)
Medium-intensity statin	30,938 (1.5)	9695 (1.3)	7344 (1.3)
Low-intensity statin	3847 (0.2)	1165 (0.2)	894 (0.2)
Ezetimibe only	49,837 (2.4)	16,552 (2.2)	12,599 (2.2)
PCSK9i (monotherapy or in combination with statin)	16,732 (0.8)	6870 (0.9)	4955 (0.9)

⁎ LDL-C was assessed among all patients with or without current LLT. ACS, acute coronary syndrome; ASCVD, atherosclerotic cardiovascular disease; CABG, coronary artery bypass graft; CHF, congestive heart failure; eGFR, estimated glomerular filtration rate; HeFH, heterozygous familial hypercholesterolemia; IS, ischemic stroke; LDL-C, low-density lipoprotein cholesterol; LLT, lipid-lowering therapy; MI, myocardial infarction; PAD, peripheral arterial disease; PCI, percutaneous coronary intervention; PCSK9i, proprotein convertase subtilisin/kexin type 9 inhibitor; SD, standard deviation; VHR, very high-risk.

### 3.2 Prevalence of VHR ASCVD criteria

**Fig. 1:**
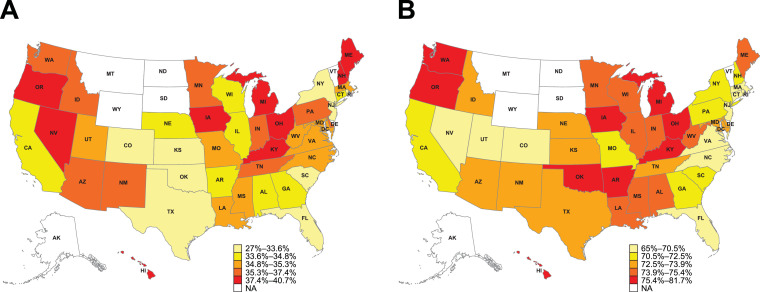
Heatmap of US geographic variations in proportions of (A) patients with a history of ≥1 major ASCVD event and (B) patients with retrospectively identified VHR ASCVD criteria. Data are age- and sex-standardized percentages. Due to low sample sizes, values for Alaska, Montana, North Dakota, South Dakota, Vermont, and Wyoming are masked. ASCVD, atherosclerotic cardiovascular disease; NA, not available; VHR, very high-risk.

Table 3 reports geographic variations in study outcomes in the states with the 10 highest and 10 lowest proportions of patients for each respective outcome; geographic variations in study outcomes across all states is reported in Supplementary Table 2. Among patients with a history of ≥1 major ASCVD event, the 2018 ACC/AHA guideline VHR ASCVD criteria were retrospectively identified in 1139,018 (74.1%) patients during the 5-year pre-index period. The most common VHR ASCVD qualifying event identified during the 5-year pre-index period was a history of MI (Table 2). In this analysis, Hawaii had the highest proportion (81.7%), while Colorado had the lowest proportion (65.0%) of patients with retrospectively identified VHR ASCVD criteria (Table 3; Supplementary Table 2; Fig. 1).

**Table 3:** Geographic variation in major ASCVD events, retrospectively identified VHR ASCVD criteria, current LLT patterns, and LDL-C ≥70 mg/dL despite current LLT with statins and/or ezetimibe (age and sex standardized).

	**Patient subgroup**					
	**≥1 major ASCVD event (%)**	**VHR ASCVD criteria(%)**	**≥1 major ASCVD event on statins and/or ezetimibe (%)**	**VHR ASCVD criteria on statins and/or ezetimibe (%)**	**≥1 major ASCVD event with LDL-C ≥70** **mg/dL (≥1.8** **mmol/L) despite statins and/or ezetimibe (%)**	**VHR ASCVD criteria with LDL-C ≥70** **mg/dL (≥1.8** **mmol/L) despite statins and/or ezetimibe (%)**
National average	33.6	72.1	48.3	49.8	57.3	58.8
State-level: 10 highest proportions	Maine (40.7)	Hawaii (81.7)	Massachusetts (55.1)	Massachusetts (57.0)	Maine (68.5)	Maine (70.6)
	Oregon (40.0)	Oregon (80.1)	Pennsylvania (54.2)	Pennsylvania (55.5)	Michigan (63.9)	Wisconsin (65.8)
	Kentucky (39.7)	Ohio (77.3)	Louisiana (54.0)	Delaware (55.5)	Wisconsin (63.9)	Michigan (64.7)
	Ohio (38.9)	Oklahoma (76.5)	Connecticut (53.8)	Louisiana (55.3)	Mississippi (61.3)	DC* (62.6)
	Michigan (38.5)	Kentucky (76.4)	Delaware (53.7)	Connecticut (55.0)	Indiana (60.9)	Arkansas (62.5)
	Hawaii (37.9)	Washington (76.4)	Missouri (52.8)	Missouri (54.0)	Arkansas (60.6)	Florida (61.8)
	Iowa (37.7)	Arkansas (76.3)	Illinois (51.8)	New Hampshire (53.6)	Minnesota (60.5)	Georgia (61.7)
	New Hampshire (37.6)	Iowa (75.7)	New Hampshire (51.5)	Rhode Island (53.6)	DC* (60.4)	Indiana (61.7)
	Nevada (37.6)	Michigan (75.5)	Rhode Island (51.2)	Illinois (53.2)	Florida (60.3)	Illinois (61.6)
	Minnesota (37.3)	Minnesota (75.4)	Ohio (51.2)	Ohio (52.9)	Illinois (59.9)	Minnesota (61.5)
State-level: 10 lowest proportions	New York (27.0)	Colorado (65.0)	Colorado (39.2)	Colorado (41.7)	Hawaii (46.9)	Hawaii (47.9)
	New Jersey (30.9)	Utah (68.1)	Arizona (42.1)	Arizona (43.7)	Colorado (47.9)	Colorado (49.9)
	Kansas (31.9)	Rhode Island (68.2)	New Mexico (42.8)	New Mexico (44.1)	Delaware (50.6)	Delaware (51.4)
	Oklahoma (32.2)	Nevada (68.8)	Oklahoma (43.4)	Nevada (45.1)	Washington (51.4)	Washington (52.7)
	Florida (32.3)	Virginia (68.9)	Nevada (43.6)	Oklahoma (45.4)	Rhode Island (53.5)	Rhode Island (54.8)
	Colorado (32.9)	Florida (69.7)	Oregon (45.3)	Florida (46.7)	New Hampshire (53.8)	Utah (55.2)
	Texas (33.0)	Massachusetts (70.3)	Florida (45.5)	Oregon (47.0)	Utah (53.8)	New Hampshire (55.4)
	South Carolina (33.2)	North Carolina (70.3)	Utah (45.7)	Maine (48.1)	North Carolina (54.0)	Arizona (55.5)
	Rhode Island (33.4)	New Jersey (70.5)	New York (46.8)	New York (48.1)	Arizona (54.5)	North Carolina (56.6)
	DC* (33.6)	Connecticut (70.5)	Maine (46.9)	Idaho (48.2)	Virginia (55.2)	Kentucky (56.6)

*DC is not a state; however it met the criteria for inclusion in the state-level analysis. ASCVD, atherosclerotic cardiovascular disease; DC, District of Columbia; LDL-C, low-density lipoprotein cholesterol; LLT, lipid-lowering therapy; VHR, very high-risk.

### 3.3 Geographic variations in LLT use

We assessed LLT use both in the 12-month and 3-month period prior to the index LDL-C date. Nationally, 64.5% of patients with a history of ≥1 major ASCVD event and 66.6% of patients with retrospectively identified VHR ASCVD criteria had ≥1 claim of any LLT use in the 12-month pre-index period (Table 2). In the 3-month pre-index period, 48.8% of patients nationally with a history of ≥1 major ASCVD event had ≥1 claim of current LLT use and, of these, 93.4% of patients received statin monotherapy with 42.2%, 44.4%, and 6.7% of them receiving high-, medium-, and low-intensity statins, respectively. Only 3.5% of patients received statin plus ezetimibe combination therapy and only 0.9% of patients received PCSK9i therapy (monotherapy or in combination with statins) (Table 2).

**Fig. 2:**
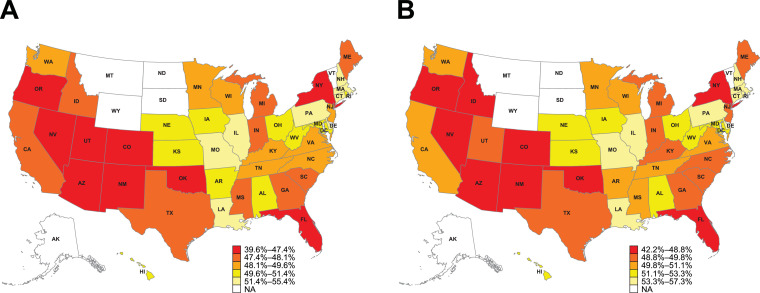
Heatmap of US geographic variations in current LLT use in (A) patients with a history of ≥1 major ASCVD event and (B) patients with retrospectively identified VHR ASCVD criteria. Data are age- and sex-standardized percentages. Due to low sample sizes, values for Alaska, Montana, North Dakota, South Dakota, Vermont, and Wyoming are masked. ASCVD, atherosclerotic cardiovascular disease; LLT, lipid-lowering therapy; NA, not available; VHR, very high-risk.

Nationally, 50.2% of patients with retrospectively identified VHR ASCVD criteria were in receipt of current LLT in the 3-month pre-index period. Of these, 42.2% of patients received high-intensity statins, and 0.9% of patients received PCSK9i (Table 2). Fig. 2 displays a heatmap of US geographic variations in age- and sex-standardized current LLT use among patients with a history of ≥1 major ASCVD event and patients with retrospectively identified VHR ASCVD criteria. The state with the highest current LLT use among patients with a history of ≥1 major ASCVD event and among patients with retrospectively identified VHR ASCVD criteria was Massachusetts, with 55.4% and 57.3% of patients, respectively, treated in the 3-month pre-index period (Fig. 2). The state with the lowest current LLT utilization among patients with a history of ≥1 major ASCVD event and among patients with retrospectively identified VHR ASCVD criteria was Colorado, with only 39.6% and 42.2% of patients, respectively, treated in the 3-month pre-index period (Fig. 2).

### 3.4 Geographic variations in persistent LDL-C elevations despite statin and/or ezetimibe use

Given that only 0.9% of both patients with ≥1 major ASCVD event and patients with retrospectively identified VHR ASCVD criteria used PCSK9i therapy, we excluded these patients while assessing persistent LDL-C elevations despite current LLT use. At the national level, after standardizing for age and sex, 57.3% and 20.3% of patients with a history of ≥1 major ASCVD event had LDL-C ≥70 mg/dL (≥1.8 mmol/L) and LDL-C ≥100 mg/dL (≥2.6 mmol/L), respectively, despite current LLT with statins and/or ezetimibe. Similarly, nationally, 58.8% and 23.7% of patients with retrospectively identified VHR ASCVD criteria had LDL-C ≥70 mg/dL (≥1.8 mmol/L) and LDL-C ≥100 mg/dL (≥2.6 mmol/L), respectively, despite current LLT with statins and/or ezetimibe.

The Central Illustration displays a heatmap of US geographic variations in the proportions of patients with a history of ≥1 major ASCVD event and patients with retrospectively identified VHR ASCVD criteria with elevated LDL-C ≥70 mg/dL (≥1.8 mmol/L) and LDL-C ≥100 mg/dL (≥2.6 mmol/L), despite current LLT with statins and/or ezetimibe. Substantial state-level variations in persistent LDL-C elevations despite current LLT with statins and/or ezetimibe were observed: Maine and Hawaii had the highest (68.5%) and lowest (46.9%) proportions, respectively, of patients with a history of ≥1 major ASCVD event with LDL-C ≥70 mg/dL (≥1.8 mmol/L) despite current LLT with statins and/or ezetimibe (Table 3; Supplementary Table 2; Central Illustration). Maine and Colorado had the highest and lowest proportions, respectively, of patients with a history of ≥1 major ASCVD event with LDL-C ≥100 mg/dL (≥2.6 mmol/L) despite current LLT with statins and/or ezetimibe (Central Illustration). A similar trend was seen among patients with retrospectively identified VHR ASCVD criteria, where Maine and Colorado had the highest and lowest proportions, respectively, of patients with LDL-C ≥100 mg/dL (≥2.6 mmol/L) despite current LLT with statins and/or ezetimibe (Central Illustration). Maine and Hawaii had the highest (70.6%) and lowest (47.9%) proportions, respectively, of patients with retrospectively identified VHR ASCVD criteria with LDL-C ≥70 mg/dL (≥1.8 mmol/L) despite current LLT with statins and/or ezetimibe (Table 3; Supplementary Table 2; Central Illustration).

## 4 Discussion

The current study provides novel real-world data on US geographic variations in LLT utilization and LDL-C levels, with this being the first study to report these outcomes at both the national and subnational level, in patients treated across US clinical practices with a history of ≥1 major ASCVD event and among patients with retrospectively identified 2018 ACC/AHA guideline VHR ASCVD criteria during a 5-year pre-index period. Nationally, the results indicate a pervasive unmet treatment need for effective lowering of LDL-C in patients with the highest-risk ASCVD. For example, 58.8% of patients with retrospectively identified VHR ASCVD criteria had LDL-C ≥70 mg/dL (≥1.8 mmol/L) despite current LLT with statins and/or ezetimibe. The current results support existing real-world studies conducted in the US that report unmet treatment needs [7], [8], [9], [10], [11], [12], [13], [14], [15]. The factors contributing to the elevated LDL-C levels observed in the current study are unclear; however, they may have included patients not taking their medications as prescribed, clinicians not regularly assessing follow-up LDL-C levels, along with a reduced focus on LDL-C levels as a secondary prevention performance measure during the study time period [21]. Broadly, the current results indicate that lipid-modifying management strategies need to be intensified; in patients with VHR ASCVD, the addition of a PCSK9i to maximally tolerated LLT was recommended in the 2018 ACC/AHA guidelines to achieve LDL-C lowering [6], with it now recognized that patients with higher cardiovascular risk derive greater benefit from PCSK9i treatment versus those with lower cardiovascular risk [22], [23], [24].

At the state level, we observed substantial geographic variations in the proportion of patients with retrospectively identified 2018 ACC/AHA guideline VHR ASCVD criteria, utilization of guideline-directed LLT, and the proportion of patients with elevated LDL-C despite current statin and/or ezetimibe use. Thus, patients’ cardiovascular risk status and likelihood to receive guideline-directed LLT varied by the state in which they were treated. For example, Massachusetts and Colorado had the highest and lowest proportions, respectively, of patients with a history of ≥1 major ASCVD event and patients with retrospectively identified VHR ASCVD criteria with current LLT use. Notably, among patients with a history of ≥1 major ASCVD event, Hawaii had the highest proportion of patients with retrospectively identified VHR ASCVD criteria, but these patients were also the “best treated”, with Hawaii having the lowest proportion of patients with retrospectively identified VHR ASCVD criteria with LDL-C ≥70 mg/dL (≥1.8 mmol/L) while treated with statins and/or ezetimibe.

The reasons for the state-level variations observed in the current study are likely to be multifactorial and complex, and it should be noted that the assessment of causal variables driving the heterogeneity was beyond the scope of the current study. However, a potential explanation for the clinical inertia observed in the states with the lowest levels of LLT utilization may include unmet educational needs for healthcare providers and patients [25], [26], [27]. For example, it has been reported that approximately 50% of healthcare providers in US clinical practices did not read the 2013 ACC/AHA blood cholesterol guidelines [25], and US clinical practices with the highest levels of statin utilization were much more likely to adopt the 2013 ACC/AHA blood cholesterol guidelines versus practices with an underutilization of statins [27]. Additionally, physician factors (e.g. beliefs about statins and cholesterol) and patient factors (e.g. statin intolerance) are also likely to play a role in explaining the state-level variations observed in the current study. For instance, a recent study demonstrated that patients treated by physicians with beliefs in statin benefit were more likely to receive 2013 ACC/AHA guideline-recommended statin intensity, whereas patients treated by clinicians expressing statin safety concerns were less likely to receive statins at a guideline-recommended intensity [28].

The current study had a number of strengths, including the very large sample size and being the first study to examine geographic variations across the US, and reporting metrics related to patients with a history of major ASCVD events and patients with retrospectively identified VHR ASCVD criteria at a subnational level. Additionally, the use of the IQVIA LRx database to supplement claims from the IQVIA PharMetrics Plus database enabled the capture of statin prescriptions that were paid out of pocket; otherwise these prescriptions may not have been captured in the traditionally used payer-sourced databases alone [29].

However, the study had a number of limitations. The study was subject to the common limitations arising from the use of insurance claims databases for clinical research such as limited generalizability to populations without health insurance, along with potential errors in coding and recording of data, and the potential for selection bias. To improve health inequity in the US and inform clinical practice and policy, future studies that assess US geographical variations in LLT patterns and LDL-C levels in ASCVD patients without health insurance are needed. Also, other than age and sex standardization, we did not adjust the data, and the potential influence of confounding variables on LLT patterns and LDL-C levels was not assessed. Lastly, the study contains limited data from the time period following the publication of the updated 2018 ACC/AHA blood cholesterol guidelines and it is likely that the results are more reflective of US clinical practice resulting from the 2013 ACC/AHA blood cholesterol guidelines published during the pre-index period of the study [30]. Consequently, the results of the study, including the low utilization rates of non-statin therapies, should be interpreted with the study time period in mind.

In conclusion, the current retrospective cohort study is the first to describe LLT patterns and LDL-C distributions in health-insured patients with major ASCVD events as retrospectively defined by the 2018 ACC/AHA guideline criteria [6] at both the national and subnational level in the US. The study highlights high rates of elevated LDL-C and pervasive underuse of LLT in patients with a history of major ASCVD events, including in the highest-risk patients with retrospectively identified VHR ASCVD criteria, with substantial state-level geographic variation. Consequently, there is an unmet treatment need for improved lipid management—through the adoption and implementation of clinical guidelines and intensification of LLT with non-statin therapy, where appropriate—in order to homogenize treatment and improve cardiovascular risk reduction across the US.

## 5 Data sharing

Qualified researchers may request data from Amgen clinical studies. Complete details are available at the following: http://www.amgen.com/datasharing.

## 6 Author contributions

Conception or design of the study; or the acquisition, analysis, or interpretation of data for the work: SJB, PBR, SN, KS, XW, RLW

Drafting the manuscript or revising it critically for important intellectual content: SJB, PBR, SN, MH, KP, KS, XW, RLW

All authors had full access to the study data, gave final approval of the version to be published and agree to be accountable for all aspects of the work.

## Declaration of Competing Interest

Seth J. Baum has served on scientific advisory boards, provided consulting, and performed clinical research for Amgen, Sanofi/Regeneron, Esperion, Akcea, AstraZeneca, Boehringer Ingelheim/Lilly, Novo Nordisk, and Gemphire; has served as a speaker for Amgen, Boehringer Ingelheim/Lilly, Novo Nordisk, and Aralez; and serves as president of Excel Medical Clinical Trials, LLC and Preventive Cardiology, Inc; spouse owns VitalRemedyMD. Kainan Sun, Xin Wang, and Rolin L. Wade are employees of IQVIA, which received consulting fees from Amgen to conduct this study. Sasikiran Nunna was an employee of IQVIA when the study was conducted and is a current employee of Bristol Myers Squibb. Pallavi B. Rane, Kiran Philip, and Mohdhar Habib are employees of Amgen Inc. and hold Amgen stock.

## Appendix Supplementary materials

Image, application 1
