# Supplementary material for: Geographic variations in lipid-lowering therapy utilization, LDL-C levels, and proportion retrospectively meeting the ACC/AHA very high-risk criteria in a real-world population of patients with major atherosclerotic cardiovascular disease events in the United States
Source: Am J Prev Cardiol. 2021 Mar 30;6:100177. doi: 10.1016/j.ajpc.2021.100177 (PMC8315617; doi:10.1016/j.ajpc.2021.100177)
Supplement: Supplementary file 1 [file mmc1.docx]

**Supplementary Material**

Supplementary Table 1. Study definitions of the 2018 ACC/AHA criteria for major ASCVD events and high-risk conditions

| **Criteria** | **Operational definition used in study** |
| --- | --- |
| **Major ASCVD events** | |
| Recent ACS (within the past 12 months) | Defined as an event of MI or unstable angina requiring hospitalization in the 12-month pre-index period. |
| History of MI (other than recent ACS event listed above) | Patients with at least 1 claim with a history of MI, assessed by MI event codes or status codes in the 5-year pre-index period (excluded recent ACS event). |
| History of IS | Patients with at least 1 event of IS assessed through IS events or IS status codes during the 5-year pre-index period. |
| Symptomatic PAD  (history of claudication with ABI <0.85, or previous revascularization or amputation) | Patients with a diagnosis of major adverse limb events (IP or OP) or patients with a CPT (IP or OP) or ICD-9/ICD-10 PCS codes (IP) related to symptomatic PAD. |
| **High-risk conditions** | |
| Age ≥65 years | Age at which the patient is ≥65 years during the index period. |
| Heterozygous familial hypercholesterolemia | Patients with one LDL-C ≥190 mg/dL (≥4.9 mmol/L) and a second LDL-C ≥190 mg/dL (≥4.9 mmol/L) or an on-treatment LDL-C ≥130 mg/dL (≥3.4 mmol/L); or at least 1 claim with an ICD-10 diagnosis code for possible/probable familial hypercholesterolemia (E78.01). |
| History of prior CABG or PCI outside of major ASCVD event(s) | a. In patients with no prior MI/IS, any revascularization event occurring during the 5-year pre-index period  b. In patients with prior MI/IS: revascularization occurring during the 30-day post-MI period was not considered. Patients with at least 1 revascularization procedure occurring outside the 30-day period after MI/IS during the 5-year pre-index period. |
| Diabetes mellitus | Assessed using diagnosis codes or anti-diabetic therapy during the 5-year pre-index period. |
| Hypertension | Assessed using diagnosis codes for hypertension during the 5-year pre-index period. |
| CKD (eGFR 15–59 mL/min/1.73 m^2^) | CKD stage III–IV assessed using ICD claims during the 5-year pre-index period (ICD-9 diagnosis codes for CKD, stage III–IV: 585.3, 585.4; ICD-10 diagnosis codes: N18.3, N18.4). |
| Current smoking | Smoking or a history of smoking during 5-year pre-index period, assessed using ICD codes for smoking. |
| Persistently elevated LDL-C (LDL-C ≥100 mg/dL [>2.6 mmol/L]) despite maximally tolerated statin therapy and/or ezetimibe | Defined as patients with 2 sequential LDL-C values >100 mg/dL (>2.6 mmol/L); or evidence of maximally tolerated or high-intensity statin within 1 year of most recent LDL-C >100 mg/dL (>2.6 mmol/L) reading; OR evidence of ezetimibe within 1 year of most recent LDL-C >100 mg/dL (>2.6 mmol/L) reading (ezetimibe did not have to overlap with statin but could); assessed in the 5-year pre-index period. |
| History of CHF | Defined as patients with at least 1 claim for CHF during the index period. ICD-9 codes for heart failure: 428.x or 428.xx; ICD-10 codes: I50.x, I50.xx. |

ABI, ankle-brachial index; ACC, American College of Cardiology; ACS, acute coronary syndrome; AHA, American Heart Association; ASCVD, atherosclerotic cardiovascular disease; CABG, coronary artery bypass graft; CHF, congestive heart failure; CKD, chronic kidney disease; CPT, Current Procedural Terminology; eGFR, estimated glomerular filtration rate; ICD, International Classification of Diseases; IP, inpatient; IS, ischemic stroke; LDL-C, low-density lipoprotein cholesterol; MI, myocardial infarction; OP, outpatient; PAD, peripheral arterial disease; PCI, percutaneous coronary intervention; PCS, Procedure Coding System.

Supplementary Table 2. Geographic variation in major ASCVD events, retrospectively identified VHR ASCVD criteria, current LLT patterns, and LDL-C ≥70 mg/dL despite current LLT with statins and/or ezetimibe (age and sex standardized).

| **State** | **≥1 major ASCVD event (%)** | **≥1 major ASCVD event on statins and/or ezetimibe (%)** | **≥1 major ASCVD event with LDL-C ≥70 mg/dL (≥1.8 mmol/L) despite statins and/or ezetimibe (%)** | **VHR ASCVD criteria**  **(%)** | **VHR ASCVD criteria on statins and/or ezetimibe (%)** | **VHR ASCVD criteria with LDL-C ≥70 mg/dL (≥1.8 mmol/L) despite statins and/or ezetimibe (%)** |
| --- | --- | --- | --- | --- | --- | --- |
| Alaska | 35.2 | 47.8 | 58.6 | 65.6 | 49.9 | N/A |
| Alabama | 34.6 | 49.8 | 56.8 | 74.3 | 50.8 | 57.8 |
| Arkansas | 34.2 | 49.5 | 60.6 | 76.3 | 50.9 | 62.5 |
| Arizona | 36.6 | 42.1 | 54.5 | 73.6 | 43.7 | 55.5 |
| California | 34.7 | 47.8 | 55.4 | 70.7 | 49.8 | 57.1 |
| Colorado | 32.9 | 39.2 | 47.9 | 65.0 | 41.7 | 49.9 |
| Connecticut | 34.5 | 53.8 | 56.0 | 70.5 | 55.0 | 57.8 |
| District of Columbia | 33.6 | 47.1 | 60.4 | 70.7 | 49.7 | 62.6 |
| Delaware | 37.2 | 53.7 | 50.6 | 73.9 | 55.5 | 51.4 |
| Florida | 32.3 | 45.5 | 60.3 | 69.7 | 46.7 | 61.8 |
| Georgia | 34.1 | 47.2 | 59.9 | 71.7 | 48.5 | 61.7 |
| Hawaii | 37.9 | 50.3 | 46.9 | 81.7 | 51.4 | 47.9 |
| Iowa | 37.7 | 50.4 | 59.5 | 75.7 | 52.3 | 61.2 |
| Idaho | 36.6 | 47.2 | 55.9 | 73.7 | 48.2 | 56.7 |
| Illinois | 34.3 | 51.8 | 59.9 | 74.3 | 53.2 | 61.6 |
| Indiana | 36.6 | 47.4 | 60.9 | 74.0 | 49.5 | 61.7 |
| Kansas | 31.9 | 50.9 | 57.2 | 73.9 | 52.6 | 58.6 |
| Kentucky | 39.7 | 48.0 | 55.6 | 76.4 | 49.0 | 56.6 |
| Louisiana | 34.8 | 54.0 | 59.7 | 74.4 | 55.3 | 61.0 |
| Massachusetts | 35.1 | 55.1 | 56.8 | 70.3 | 57.0 | 57.8 |
| Maryland | 34.8 | 49.6 | 55.6 | 73.6 | 51.4 | 57.0 |
| Maine | 40.7 | 46.9 | 68.5 | 74.5 | 48.1 | 70.6 |
| Michigan | 38.5 | 47.5 | 63.9 | 75.5 | 49.5 | 64.7 |
| Minnesota | 37.3 | 48.6 | 60.5 | 75.4 | 50.8 | 61.5 |
| Missouri | 35.1 | 52.8 | 57.1 | 72.4 | 54.0 | 58.6 |
| Mississippi | 34.8 | 47.7 | 61.3 | 74.5 | 49.7 | 61.3 |
| Montana | 33.7 | 44.7 | 57.8 | 73.6 | 49.0 | 61.9 |
| North Carolina | 34.9 | 48.2 | 54.0 | 70.3 | 49.5 | 56.6 |
| North Dakota | 35.0 | 41.7 | 71.8 | 76.7 | 45.1 | 71.1 |
| Nebraska | 34.6 | 50.2 | 56.6 | 72.6 | 51.5 | 57.6 |
| New Hampshire | 37.6 | 51.5 | 53.8 | 71.3 | 53.6 | 55.4 |
| New Jersey | 30.9 | 47.8 | 56.9 | 70.5 | 49.2 | 58.2 |
| New Mexico | 35.7 | 42.8 | 56.8 | 72.8 | 44.1 | 60.2 |
| Nevada | 37.6 | 43.6 | 59.4 | 68.8 | 45.1 | 60.7 |
| New York | 27.0 | 46.8 | 58.1 | 71.5 | 48.1 | 59.6 |
| Ohio | 38.9 | 51.2 | 58.4 | 77.3 | 52.9 | 59.7 |
| Oklahoma | 32.2 | 43.4 | 58.4 | 76.5 | 45.4 | 60.0 |
| Oregon | 40.0 | 45.3 | 58.9 | 80.1 | 47.0 | 59.0 |
| Pennsylvania | 36.4 | 54.2 | 56.4 | 72.4 | 55.5 | 57.8 |
| Rhode Island | 33.4 | 51.2 | 53.5 | 68.2 | 53.6 | 54.8 |
| South Carolina | 33.2 | 47.6 | 56.6 | 72.0 | 49.4 | 58.4 |
| South Dakota | 37.4 | 44.7 | 69.3 | 81.3 | 46.9 | 72.8 |
| Tennessee | 36.7 | 49.3 | 57.9 | 73.4 | 50.7 | 59.3 |
| Texas | 33.0 | 47.7 | 56.8 | 73.0 | 49.1 | 58.4 |
| Utah | 35.0 | 45.7 | 53.8 | 68.1 | 48.7 | 55.2 |
| Virginia | 34.9 | 48.9 | 55.2 | 68.9 | 50.8 | 57.0 |
| Vermont | 36.6 | 46.4 | N/A | 72.6 | 47.1 | N/A |
| Washington | 37.2 | 48.0 | 51.4 | 76.4 | 49.9 | 52.7 |
| Wisconsin | 34.7 | 47.9 | 63.9 | 74.2 | 50.1 | 65.8 |
| West Virginia | 34.9 | 49.9 | 57.0 | 75.1 | 51.0 | 58.4 |
| Wyoming | 33.5 | 43.7 | N/A | 70.7 | 43.8 | N/A |

ASCVD, atherosclerotic cardiovascular disease; DC, District of Columbia; LDL-C, low-density lipoprotein cholesterol; LLT, lipid-lowering therapy; N/A, not available; VHR, very high-risk.


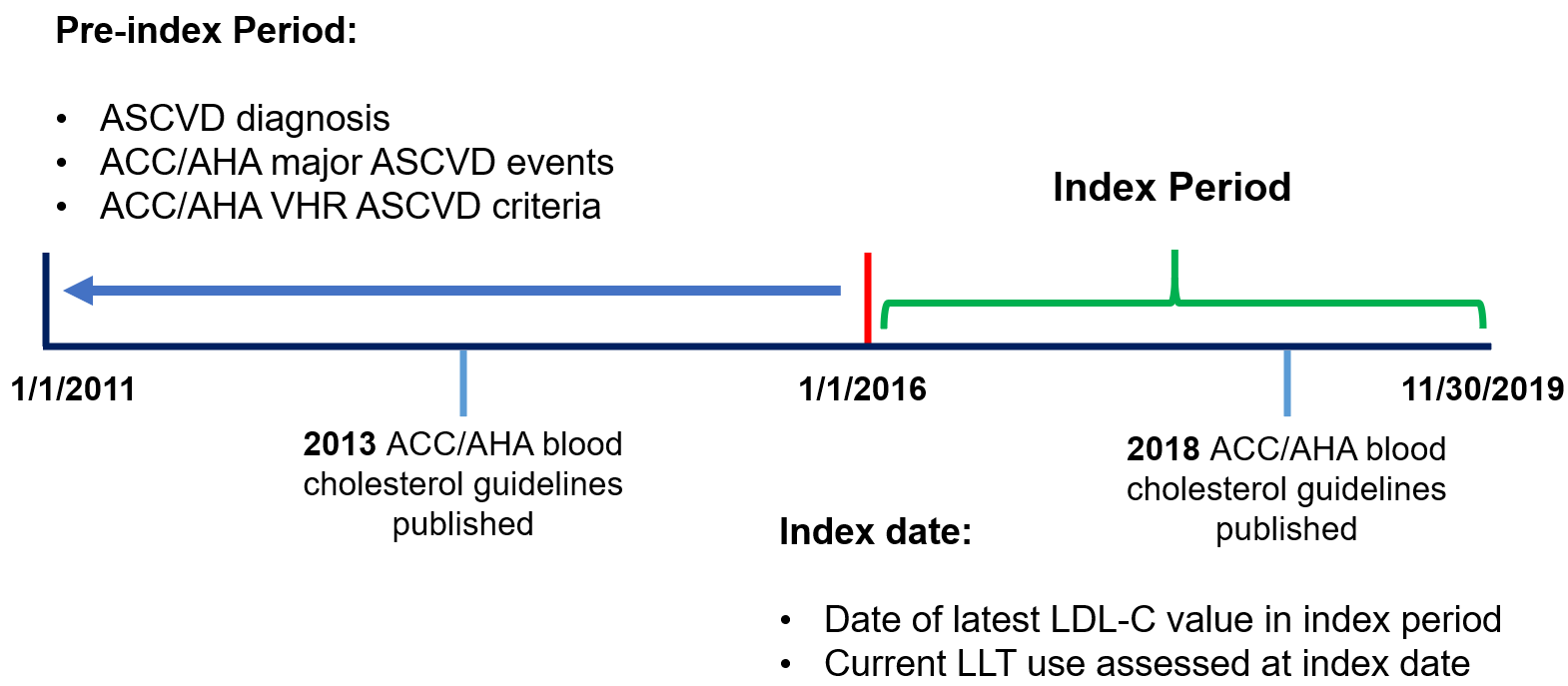


Supplementary Figure 1. Study design.

ACC, American College of Cardiology; AHA, American Heart Association; ASCVD, atherosclerotic cardiovascular disease; LDL-C, low-density lipoprotein cholesterol; LLT, lipid-lowering therapy; VHR, very high-risk.
